# Novel Alzheimer’s disease risk variants identified based on whole-genome sequencing of *APOE* ε4 carriers

**DOI:** 10.1038/s41398-021-01412-9

**Published:** 2021-05-19

**Authors:** Jong-Ho Park, Inho Park, Emilia Moonkyung Youm, Sejoon Lee, June-Hee Park, Jongan Lee, Dong Young Lee, Min Soo Byun, Jun Ho Lee, Dahyun Yi, Sun Ju Chung, Kye Won Park, Nari Choi, Seong Yoon Kim, Woon Yoon, Hoyoung An, Ki woong Kim, Seong Hye Choi, Jee Hyang Jeong, Eun-Joo Kim, Hyojin Kang, Junehawk Lee, Younghoon Kim, Eunjung Alice Lee, Sang Won Seo, Duk L. Na, Jong-Won Kim

**Affiliations:** 1 Clinical Genomics Center, Samsung Medical Center, Seoul, South Korea; 2 Center for Precision Medicine, Gangnam Severance Hospital, Yonsei University College of Medicine, Seoul, South Korea; 3 Department of Pathology, Gangnam Severance Hospital, Yonsei University College of Medicine, Seoul, South Korea; 4 Department of Health Sciences and Technology, Samsung Advanced Institute for Health Sciences and Technology, Sungkyunkwan University, Samsung Medical Center, Seoul, South Korea; 5 Precision Medicine Center, Seoul National University Bundang Hospital, Seongnam, South Korea; 6 Research Institute for Future Medicine, Samsung Medical Center, Seoul, South Korea; 7 Subtropical Livestock Research Institute, National Institute of Animal Science, RDA, Jeju, South Korea; 8 Department of Neuropsychiatry, Seoul National University Hospital & Department of Psychiatry, Seoul National University College of Medicine, 101 Daehak-ro, Jongno-gu, Seoul, South Korea; 9 Department of Neuropsychiatry, Seoul National University Bundang Hospital, Seongnam, South Korea; 10 Department of Psychiatry, National Center for Mental Health, Seoul, South Korea; 11 Institute of Human Behavioral Medicine, Medical Research Center Seoul National University, Seoul, South Korea; 12 Department of Neurology, Asan Medical Center, University of Ulsan College of Medicine, Seoul, South Korea; 13 Department of Psychiatry, Asan Medical Center, University of Ulsan College of Medicine, Seoul, South Korea; 14 Department of Neuropsychiatry, St. Andrew’s Hospital, Icheon, South Korea; 15 Department of Brain and Cognitive Science, Seoul National University College of Natural Sciences, Seoul, South Korea; 16 Department of Psychiatry, Seoul National University, College of Medicine, Seoul, South Korea; 17 Department of Neurology, Inha University School of Medicine, Incheon, South Korea; 18 Department of Neurology, Ewha Womans University Seoul Hospital, Ewha Womans University School of Medicine, Seoul, South Korea; 19 Department of Neurology, Pusan National University Hospital and Biomedical Research Institute, Pusan National University School of Medicine, Busan, South Korea; 20 Division of Supercomputing, KISTI, Daejeon, South Korea; 21 Division of Genetics and Genomics, Boston Children’s Hospital, Harvard Medical School, Boston, MA USA; 22 Broad Institute of MIT and Harvard, Cambridge, MA USA; 23 Department of Neurology, Sungkyunkwan University School of Medicine and Neuroscience Center, Samsung Medical Center, Seoul, South Korea; 24 Department of Laboratory Medicine and Genetics, Samsung Medical Center, Sungkyunkwan University School of Medicine, Seoul, South Korea

**Keywords:** Comparative genomics, Clinical genetics

## Abstract

Alzheimer’s disease (AD) is a progressive neurodegenerative disease associated with a complex genetic etiology. Besides the apolipoprotein E ε4 (*APOE* ε4) allele, a few dozen other genetic loci associated with AD have been identified through genome-wide association studies (GWAS) conducted mainly in individuals of European ancestry. Recently, several GWAS performed in other ethnic groups have shown the importance of replicating studies that identify previously established risk loci and searching for novel risk loci. *APOE*-stratified GWAS have yielded novel AD risk loci that might be masked by, or be dependent on, *APOE* alleles. We performed whole-genome sequencing (WGS) on DNA from blood samples of 331 AD patients and 169 elderly controls of Korean ethnicity who were *APOE* ε4 carriers. Based on WGS data, we designed a customized AD chip (cAD chip) for further analysis on an independent set of 543 AD patients and 894 elderly controls of the same ethnicity, regardless of their *APOE* ε4 allele status. Combined analysis of WGS and cAD chip data revealed that SNPs rs1890078 (*P* = 6.64E−07) and rs12594991 (*P* = 2.03E−07) in *SORCS1* and *CHD2* genes, respectively, are novel genetic variants among *APOE* ε4 carriers in the Korean population. In addition, nine possible novel variants that were rare in individuals of European ancestry but common in East Asia were identified. This study demonstrates that *APOE*-stratified analysis is important for understanding the genetic background of AD in different populations.

## Introduction

Alzheimer’s disease (AD) is the most common form of dementia, accounting for 60–70% of all cases^1^. In the United States, the prevalence of AD in people aged 65 years and older is forecast to increase from 4.7% in 2010 to 13.8% in 2050^2,3^. Likewise, in South Korea, the prevalence of AD in people aged 65 years and older is forecast to increase from 9.95% in 2017 to 16.09% in 2050^4^.

Late-Onset Alzheimer’s disease (LOAD), the non-Mendelian form of AD, accounts for >99% of AD and is highly heritable (estimates of 58–79%), with complex genetic etiology^5,6^. The *APOE* ε4 allele is the strongest genetic risk factor for LOAD, with a population attributable fraction of approximately 30–35%^7^. The search for additional genetic risk factors through genome-wide association studies (GWAS) conducted mainly in European populations have yielded a few dozen genetic loci, beyond *APOE* ε4, associated with LOAD^8–12^. However, the genetic liability of AD by *APOE* and GWAS findings is estimated to be only 24–33%, which is not enough to explain the 58–79% heritability for AD revealed by twin studies^5,6^.

The effects of many risk loci differ across ethnic groups. For example, variants in *ABCA7* have greater effects on AD risk for individuals of African–American ancestry than on those of European ancestry^13^. One main reason for this is differences in frequencies of risk alleles in different ethnic groups, which affects calculations of odds ratios and statistical significance. Many AD loci can be revealed by focusing on ethnic groups other than European. For example, GWAS in non-European populations have identified novel genetic risk factors associated with AD, such as *SORL1* from Japanese studies and *ACE* from Israeli–Arabs^14,15^. Morris et al. recently showed that racial differences are correlated with molecular biomarkers, such as the levels of t-tau and p-tau_181_ proteins in cerebrospinal fluid of AD patients^16^. Several GWAS where individuals were stratified based on *APOE* status have also led to the identification of novel loci^17,18^. For example, *KANSL1* region on chromosome 17 near *MAPT* was identified using only *APOE* ε4 negative (*APOE* ε4-) samples in the International Genomics of Alzheimer’s Project (IGAP)^17^. Novel associations were also identified with variants in *ISYNA1*, *OR8G5*, *IGHV3-7*, and *SLC24A3*, among *APOE* ε4 carriers^19^. We therefore rationalized that we would be more likely to find novel genetic variants by focusing on a subgroup of individuals of Korean ethnicity that was stratified based on *APOE* ε4 allele status.

The goal of the present study was to identify genetic markers that are significantly associated with AD among *APOE* ε4 carriers in the Korean population by conducting the following analyses: (1) identifying possible candidate markers from a dataset of 500 whole-genome sequencing (WGS) comprised of 331 AD cases and 169 controls; (2) designing a customized genotyping AD chip (cAD chip) containing our WGS candidates and previously known genetic variants associated with AD; and (3) validating significant genetic markers in independent datasets from the Korean population by using the cAD chip.

## Materials and methods

### Sample information

In total, 331 AD patients and 169 control participants aged 70 years or older, with normal cognitive ability, were recruited as a discovery set. All 500 participants used in the discovery set were of Korean descent and bore the *APOE* ε4 positive (*APOE* ε4+) allele. A separate set of 1915 participants, also of Korean descent, including 287 bearing the *APOE* ε4+ allele, were recruited as a validation set. All participants were enrolled on the basis of the medical records written by Geriatric neuropsychiatrists with expertise in dementia research. Each participant’s cognitive status was diagnosed based on a medical history assessment, neuropsychological test, and medical imaging with magnetic resonance imaging or amyloid positron emission tomography data. All study protocols were reviewed and approved by the relevant Institutional Review Boards at the Samsung Medical Center, Seoul, South Korea.

### Whole-genome sequencing analysis

WGS data were generated using the Illumina HiSeq X Ten platform (Illumina, San Diego, CA, USA). Library construction and 150-bp paired-end sequencing were performed according to the manufacturer’s instructions. The average depth showed 18× coverage of the whole genome. Sequencing fastq data were aligned to the reference genome, hg19, with the decoy sequence using the BWA-mem algorithm implemented in BWA 0.7.10^20^. Duplicate reads were removed using Picard 1.1 (https://broadinstitute.github.io/picard/). Variant detection was performed using GenomeAnalysisTK-3.3–0^21^. Variant annotation was conducted using ANNOVAR^22^ for refGene with dbSNP 147; population frequency was assessed with gnomAD^23^. In total, 22,526,987 variants (SNPs and indels) were obtained from the raw variant call set. We applied the following hard filter criteria: (1) exclude multi-allelic variants; (2) include variants with total read depth over six; (3) include variants with alternative read depth over three, (4) exclude segmental duplication variants, and (5) exclude very rare variants with minor allele frequency (MAF) less than 0.1%.

### Design of the customized genotyping AD chip

To validate our candidates, we designed a cAD chip (based on an Axiom^®^ myDesign GW genotyping array; Thermo Fisher Scientific, Waltham, MA, USA) that would allow precise genetic characterization of samples in the context of genetic variants associated with AD. Candidates available on the cAD chip were taken from three primary sources: variants in AD-related databases, variants reported in the literature, and candidates from the WGS analysis of the discovery set.

For known AD-related candidates, we referenced the AlzGene database (http://www.alzgene.org/), the NHGRI-EBI GWAS catalog (www.ebi.ac.uk/gwas/), the single nucleotide polymorphism database (dbSNP; https://www.ncbi.nlm.nih.gov/SNP/), the Human Gene Mutation Database (HGMD; http://www.hgmd.cf.ac.uk/), as well as other candidates reported in the literature. From the AlzGene database, we selected 1448 genetic variants associated with AD^24^. A systematic review of published literature was performed to include variants known to be involved in AD. We used the NHGRI-EBI GWAS catalog curated from published GWAS since 2008^25^. The GWAS catalog data were downloaded from the University of California, Santa Cruz table browser, and candidates were selected that included the word “Alzheimer” for disease and trait type. The AD chip also contains the IGAP data released from the 2013 meta-analysis results for AD^8^. We selected 4131 variants with *P* values less than 0.00001 in the stage 1 results of 17,008 AD cases and 37,154 controls. Meta-analysis results included an independent set of 8572 AD cases and 11,312 controls with combined *P* values. We also considered *APOE*-stratified GWAS results re-analyzed from IGAP consortium data for *APOE* ε4+ (10,352 cases and 9207 controls) and *APOE* ε4- (7184 cases and 26,968 controls) subgroups. An additional 1127 candidates were derived from the original article published by Jun et al.^17^. Exonic variants located in *APP*, *PSEN1*, and *PSEN2*, known to cause autosomal dominant early-onset AD, were included from dbSNP 147 and HGMD (Supplementary Table 1).

To prioritize variants from WGS analysis of the discovery set, we gathered five types of candidate groups: coding variants, non-coding variants, case-only, control-only, and expression quantitative trait loci (eQTL) groups. Supplementary Table 1 shows the summarized information of the candidate variants in the cAD chip. First, to identify association signals from coding and non-coding region candidates, we conducted an association analysis of whole-genome variants by comparing AD samples versus controls for 500 WGS samples. We selected candidates with *P* values less than 0.05 for variants within a coding region and less than 0.001 for variants within a non-coding region. This led to the inclusion of 1396 coding variants and 29,606 non-coding variants on the chip. Second, to include rare variants, we selected variants that appeared in the above two samples in AD cases or controls. We identified 2357 AD case-only variants and 327 control-only variants. Finally, we added 570 known eQTL variants expressed in brain tissues from the HaploReg v4.1 database that mapped to the non-coding variants from the 500 WGS data^26^.

### Chip genotyping and QCs

We genotyped samples from 1915 participants using the cAD chip for replication of WGS analysis and performance evaluation of the customized chip. All DNA samples were extracted from whole blood. Our genotype calling workflow on the Affymetrix^®^ GeneTitan^®^ platform with the Axiom^®^ myDesign GW genotyping array was performed according to the Axiom^®^ 2.0 reagent kit protocol for 384 samples (Affymetrix^®^/Thermo Fisher Scientific). Fifty samples previously used for WGS analysis were also included to evaluate the cAD chip performance in order to compare the WGS results for the corresponding SNPs that overlapped both datasets. One sample with experimental error was excluded from the genotyping analysis. Raw CEL data files generated by the GeneTitan^®^ were imported into the Axiom^®^ Analysis Suite Software (version 3.0, Affymetrix/Thermo Fisher Scientific), and analysis was performed using Affymetrix Best Practices Workflow with the default threshold settings. All five plates passed plate QC; 19 samples with failing dish QC < 0.82, 70 samples with calling QC call rates < 0.97, and five samples determined to be technical duplicates were excluded from the analysis. The average call rate for the passing samples was 99.57%. We also reviewed the individual clinical charts for each patient and adopted only AD patients and candidates with normal cognition as controls, excluding 255 samples from participants with mild cognitive impairment and samples from candidates less than 55 years old. We excluded 78 samples with discordant clinical information regarding *APOE* status and sex and computed results based on the cAD chip. After genotyping, we also applied high-accuracy variant QC that excluded missing genotype rates > 5%, significant deviations from Hardy–Weinberg equilibrium *P* < 0.000001 in controls, and monomorphic variants, and included the best-recommended variants from the Axiom^®^ Analysis Suite Software. In total, 26,242 variants in 1437 samples, 543 AD cases, and 894 healthy controls including 190 AD cases and 97 controls from the *APOE* ε4 carriers, were used for the final analysis.

### Statistical analysis

All statistical analyses, including association analysis, were performed using PLINK version 1.09^27^ and the statistical software R (R Foundation for Statistical Computing, Austria). LocusZoom software was used to depict candidate regions in detail^28^. Linkage disequilibrium (LD) information from the 1000 Genomes Project (1KGP) and functional annotation of non-coding variants were obtained from HaploReg v4.1^26^.

## Results

### Identification of candidate variants by using WGS in a Korean population

**Fig. 1:**
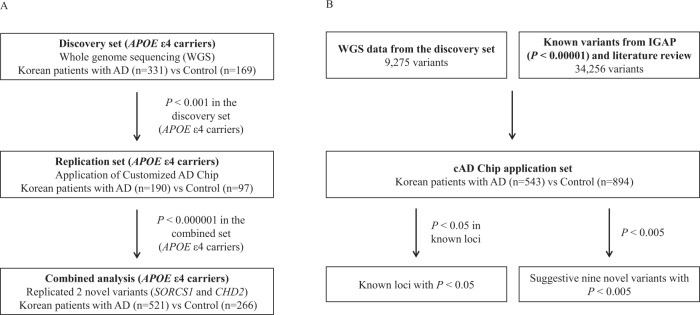
Schematic workflows. The schematic workflow shows the procedures for detecting novel genetic variants associated with Alzheimer’s Disease (AD) in a Korean population of *APOE* ε4 carriers (**A**) and for assessing the reproducibility of previously reported AD loci and suggestive novel loci shown to be population-specific (**B**). cAD customized genotyping AD chip, IGAP International Genomics of Alzheimer’s Project.

We conducted WGS of AD patients and matched controls in a Korean population who were *APOE* ε4 carriers, to find genetic variants dependent on *APOE* ε4 allele status, which is highly associated with AD pathogenesis. Our WGS analysis identified 22,526,987 variants from an *APOE* ε4 carrier dataset comprised of 331 AD patients and 169 controls. Results from all the samples confirmed that there was an ethnic overlap in genetic background with the East Asian population (JPT + CHB population) and that there was no population stratification in our discovery set (Supplementary Fig. 1). To overcome limitations in sample size and lack of power to reach genome-wide significance, we replicated 34,256 variants identified by WGS using a cAD chip (Supplementary Table 1). The chip included known variants associated with AD collected from various sources such as the IGAP. Clinical and demographic characteristics are shown in Supplementary Table 2. Our discovery and validation procedures are schematized in Fig. 1.

### Characterization of cAD chip content and concordance with WGS

In total, the AD chip contains 41,735 autosomal variants and 745 X-chromosome variants. Of these, 40,395 (95.1%) variants were found in the single nucleotide polymorphism database (dbSNP) build 147. In addition, 2113 small insertion or deletion variants were also included in the cAD chip. Annotation using ANNOVAR showed that the cAD chip contained 4855 exonic, 14,155 intronic, and 20,416 intergenic variants (Supplementary Tables 3 and 4).

To evaluate the accuracy of the cAD chip, we compared 50 samples randomly selected from the WGS discovery set. All samples exhibited high call rates (average = 99.59%) and dish quality control (QC) (average = 0.97) in the results generated by the cAD chip analysis. The average concordance rate of 25,416 autosomal genotype calls that overlapped both the cAD chip and WGS genotypes in 50 samples was 99.29% (range = 98.47–99.69%). Considering SNPs exclusively, we observed high concordance rates ranging from 98.50% to 99.70% (average = 99.31%).

### Known genetic variants associated with AD in cAD chip application set

**Fig. 2:**
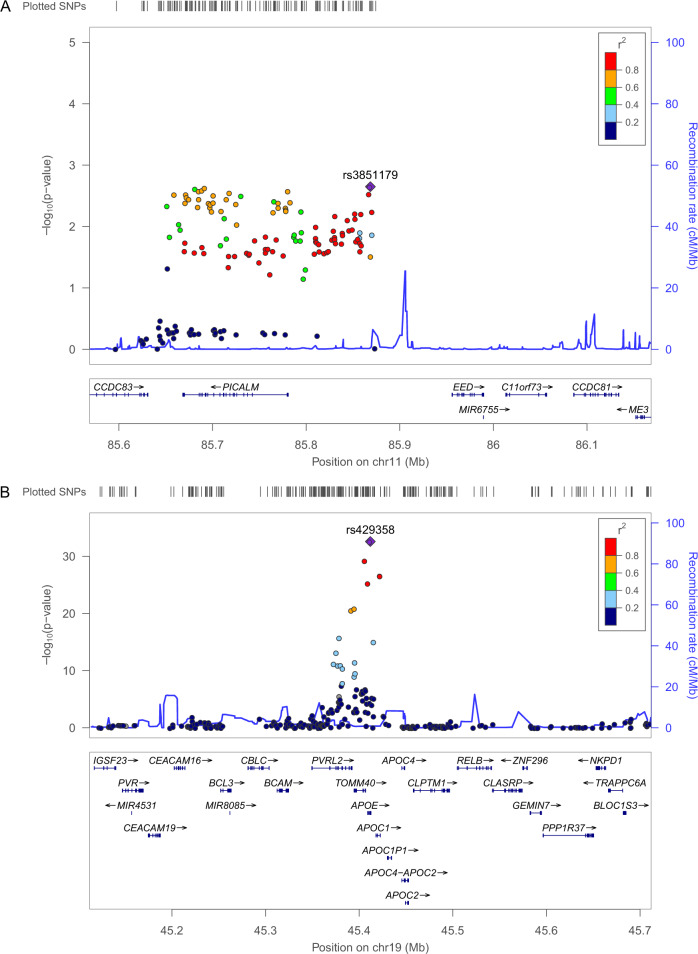
Regional plot of known loci (11q14.2; *PICALM* and 19q13.32; *APOE*) that are significantly associated with Alzheimer’s disease (AD) in the cAD chip application set. The figures show the regional association plot of known representative loci, (**A**) 11q14.2; *PICALM* and (**B**) 19q13.32; *APOE*, in the cAD chip application set (*n* = 1,437). The purple shaded diamond shape represents rs3851179 and rs429358, which are the most significant SNPs in 11q14.2 (**A)** and 19q13.32 (**B**), respectively. The blue line indicates the recombination rate, while filled color represents the linkage disequilibrium score based on *r*^2^ values estimated from the 1000 genome Nov 2014 ASN data.

We first examined the reproducibility of previously reported AD loci in 543 AD cases and 894 controls in the cAD chip application set (Table 1). We assessed 40 susceptibility loci previously reported to be associated with AD from four GWAS published by Andrews et. al.^11^. We identified 12 loci: *CR1*, *BIN1*, *CD2AP*, *GPR141*, *PILRA*, *EPHA1*, *CLU*, *PICALM, SORL1*, *SLC24A2*, *ABCA7*, and *APOE*, exhibiting significant association (*P* value < 0.05) in the cAD chip analysis. The *APOE* region in particular [best SNP: rs429358, *P* = 2.59 × 10^-33^, OR = 4.81 (95% CI: 3.76–6.16)] exhibited the highest significant association signal in the Korean population. *APOE* ε4 is the strongest genetic risk factor for AD, that has been identified in recent decades. The *PICALM* region [best SNP: rs3851179, *P* = 2.25 × 10^−3^, OR = 0.78 (95% CI: 0.67–0.92)] was also replicated in our data and showed an effect size similar to that observed in the IGAP study (Fig. 2). Our best SNP (rs3851179) in the *PICALM* region is in high LD (*r*^2^ = 1 based on the 1KGP phase 1 population) with the SNP previously reported as the best level of association (rs10792832) with AD in IGAP results^8^. Although the *EPHA1* region showed a significant association [best SNP: rs11771145, *P* = 4.75 × 10^−3^, OR = 1.24 (95% CI: 1.06–1.44)], the allelic effect of the same SNP from a previous report was in the opposite direction [OR: 1.24 (95% CI: 1.06–1.44) vs. 0.90 (95% CI: 0.88–0.93)]. Our study corroborated the association of known AD variants reported by Lambert et al. in a Korean population^8^. *BIN1* [rs6733839, *P* = 0.049, OR = 1.17 (95% CI: 1.00–1.36)], *CLU* [rs9331896, *P* = 0.021, OR = 0.81(95% CI: 0.68–0.97)], *PICALM* [rs10792832, *P* = 0.0029, OR = 0.79(95% CI: 0.67–0.92)], and *ABCA7* [rs4147929, *P* = 0.045, OR = 1.18 (95% CI: 1.01–1.38)] showed significant association with the same risk allele and direction in cAD chip results. However, we observed that not all SNPs showing the best level of association in the IGAP study were not the most strongly associated SNPs in the loci analyzed in our dataset. One such example is rs4732729 [*P* = 0.013, OR = 0.80 (95% CI: 0.67–0.96)], located in *CLU*, which exhibits a more significant association than rs9331896. Notably, the allelic effect (minor allele) of rs4732729 was opposite to that of the IGAP dataset [OR: 0.80 (95% CI: 0.67–0.96) vs. OR: 1.12 (95% CI: 1.07–1.16)]. This discrepancy may be attributed to the different LD relationships between rs9331896 and rs4732729 (*r*^2^ = 0.96 based on 1KGP phase 1 ASN population and *r*^2^ = 0.33 based on the 1KGP phase 1 EUR population) in different population. This suggests that it is important to interpret the results while considering the population LD structure. Ethnic comparison of known variants reported from IGAP between Korean and European populations showed a low correlation coefficient (r = 0.216, *P* < 2.2E−16; Supplementary Fig. 2). Taken together, our results suggest that there are ethnic differences in associated SNPs at loci related to AD among populations, even when shared risk variants are associated with AD.

**Table 1:** List of known loci significantly associated with AD in the customized genotyping AD chip application set (*n* = 1437).

					Total group (*n* = 1437)				*APOE* ε4 carriers (*n* = 287)				*APOE* ε4 non-carriers (*n* = 1150)				IGAP study^a^		
Locus	SNP^b^	Position^c^	Nearby genes to each SNP^d^	Minor/major allele	MAF in cases	MAF in controls	*P* value^e^	OR (CI, 95%)	MAF in cases	MAF in controls	*P* value^e^	OR (CI, 95%)	MAF in cases	MAF in controls	*P* value^e^	OR (CI, 95%)	Non-effect/effect alleles	Beta (SE)^f^	*P* value^e^
1q32.2	rs12743911	207816480	*CR1,CR1L*	T/C	0.45	0.41	3.38.E−02	1.18 (1.02–1.38)	0.44	0.44	9.78.E–01	1.00 (0.70–1.41)	0.45	0.4	3.18.E−02	1.22 (1.02–1.46)	C/T	0.06 (0.02)	3.24.E−04
2q14.3	rs13025765	127886233	*BIN1,CYP27C1*	T/C	0.36	0.31	2.75.E−03	1.27 (1.08–1.49)	0.37	0.34	5.08.E–01	1.13 (0.79−1.63)	0.36	0.3	1.04.E−02	1.27 (1.05–1.53)	C/T	0.13 (0.02)	7.87.E−13
6p12.3	rs12202615	47618171	*CD2AP,ADGRF2*	C/T	0.14	0.094	3.54.E−04	1.50 (1.18–1.91)	NA	NA	NA	NA	0.14	0.09	6.45.E−04	1.56 (1.19–2.05)	T/C	0.08 (0.02)	4.68.E−06
7p14.1	rs10261983	37885121	*GPR141,NME8*	C/T	0.45	0.50	1.16.E−02	0.82 (0.71–0.96)	0.43	0.51	7.69.E–02	0.73 (0.51–1.03)	0.46	0.50	1.21.E−01	0.87 (0.73–1.04)	C/T	−0.07 (0.02)	1.28.E−05
7q22.1	rs2405442	99971313	*PILRA*	C/T	0.44	0.40	2.80.E−02	1.18 (1.02–1.38)	0.44	0.42	7.47.E−01	1.06 (0.75−1.50)	0.44	0.39	4.12.E−02	1.20 (1.00–1.44)	C/T	−0.07 (0.02)	4.23.E−05
7q35	rs11771145	143110762	*EPHA1-AS1*	G/A	0.51	0.46	4.75.E−03	1.24 (1.06–1.44)	0.51	0.42	4.73.E−02	1.43 (1.00−2.02)	0.51	0.46	2.90.E−02	1.21 (1.01–1.45)	G/A	−0.1 (0.02)	8.76.E−10
8p21.1	rs4732729	27461492	*CLU*	C/A	0.22	0.26	1.32.E−02	0.80 (0.67–0.96)	0.22	0.28	1.45.E−01	0.75 (0.50–1.11)	0.22	0.26	3.79.E−02	0.80 (0.65–0.99)	C/A	0.11 (0.02)	1.26.E−09
11q14.2	rs3851179	85868640	*PICALM,EED*	T/C	0.34	0.4	2.25.E−03	0.78 (0.67–0.92)	0.35	0.41	1.66.E−01	0.77 (0.54−1.11)	0.34	0.4	6.35.E−03	0.78 (0.64–0.93)	C/T	−0.13 (0.02)	2.84.E−15
11q24.1	rs4936632	121335728	*SORL1*	A/G	0.44	0.48	1.26.E−02	0.83 (0.71–0.96)	0.43	0.5	1.18.E−01	0.77 (0.54−1.09)	0.44	0.48	5.32.E−02	0.84 (0.70–1.01)	G/A	−0.02 (0.02)	1.49.E−01
14q32.12	rs7154691	92934699	*SLC24A4*	A/G	0.51	0.47	3.31.E−02	1.18 (1.01–1.37)	0.47	0.53	1.68.E−01	0.78 (0.55−1.11)	0.50	0.47	1.75.E−01	1.13 (0.95–1.35)	G/A	0.08 (0.02)	1.34.E−05
19p13.3	rs4147911	1047687	*ABCA7*	G/C	0.37	0.33	1.77.E−02	1.22 (1.04–1.43)	0.35	0.34	9.47.E−01	1.01 (0.70–1.46)	0.39	0.33	6.03.E−03	1.30 (1.08–1.57)	C/G	0.19 (0.03)	1.25.E−08
19q13.32	rs429358	45411941	*APOE*	C/T	0.22	0.056	2.59.E−33	4.81 (3.76–6.16)	0.36	0.48	1.80.E−06	0.61 (0.43–0.87)	NA	NA	NA	NA	T/C	1.35 (0.03)	6.70.E−536

*AD* Alzheimer’s disease, *MAF* minor allele frequency, *OR* odds ratio, *CI* confidence interval, *SNP* single nucleotide polymorphism, *NA* not available.

^a^Non-effect/effect alleles, Beta and *P* values in the International Genomics of Alzheimer’s Project (IGAP).

^b^The most significant SNPs associated with AD in the total sample set within each locus.

^c^Physical position based on human reference genome build hg19 (GRCh37).

^d^The nearest gene to each SNP is underlined.

^e^*P* values were calculated using Cochran-Armitage trend test.

^f^Overall estimated effect size and standard error for the effect allele.

### Novel variants associated with AD in *APOE* ε4 carriers

**Fig. 3:**
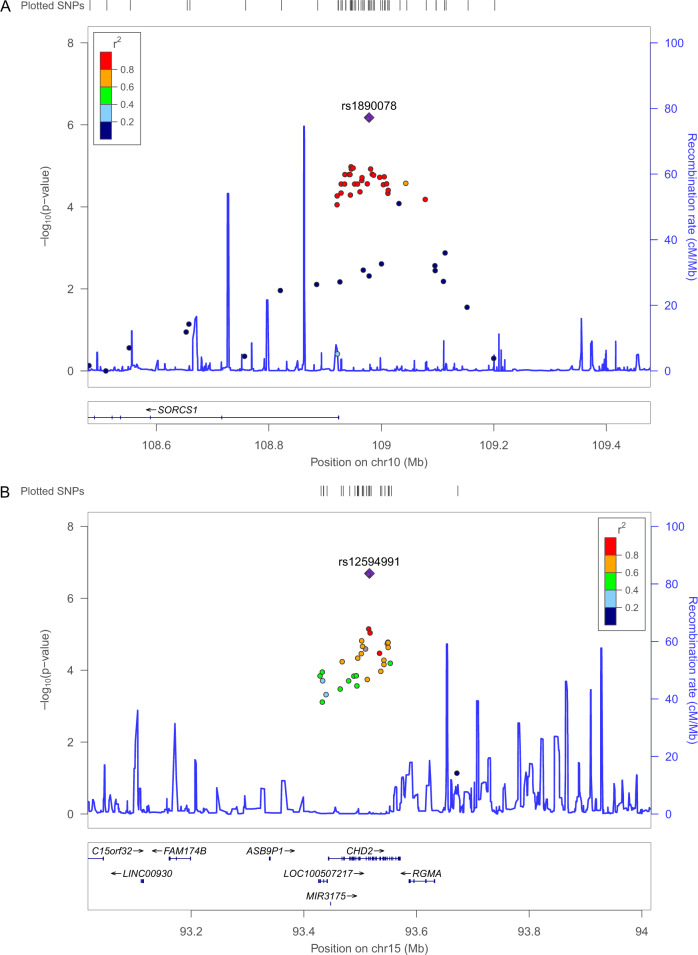
Regional plot of novel candidate variants significantly associated within *APOE* ε4 carriers. The figures show the regional association plot of known representative loci, **(A**) 10q25.1; *SORCS1* and (**B**) 15q26.1; *CHD2*, in the cAD chip application set with a combined *P* value < 0.000001. The purple shaded diamond shape represents rs1890078 and rs12594991, which are the most significant SNPs in 10q25.1 (**A**) and 15q26.1 (**B**), respectively. The blue line indicates the recombination rate, while filled color represents the linkage disequilibrium score based on *r*^2^ values estimated from the 1000 genome Nov 2014 ASN data.

We identified two novel AD-associated SNPs in *APOE* ε4 carriers with a discovery *P* value < 10^−3^, a replication *P* value of 0.05, and a combined *P* value < 10^−6^ (Table 2). One intergenic SNP between *SORCS1* and *LINC01435* [rs1890078; *P* = 6.64 × 10^−7^, OR = 0.43 (95% CI: 0.30–0.61)] showed higher MAF allele, C in control samples as a protective effect associated with reduced risk of AD (Fig. 3). Located in the intron of *CHD2*, SNP rs12594991, showed higher frequency of the minor allele, A in AD cases. The frequency of the A allele in rs12594991 in the European population (0.52) is much higher than in the Asian population (0.15). Two SNPs (rs1890078 and rs12594991) did not exhibit any significant association with AD in the *APOE* ε4 non-carriers (Supplementary Table 5).

**Table 2:** List of novel candidate variants significantly associated within *APOE* ε4 carriers (Combined *P* value < 0.000001).

Chromosome	SNP	Position^a^	Assigned genes^b^	Functional refGene	Minor/major allele	Cohort	MAF in cases	MAF in controls	*P* value^c^	OR (CI, 95%)	East Asian frequency^d^	European frequency (non-Finnish)^d^
10	rs1890078	108978236	*SORCS1,LINC01435*	intergenic	C/T	Discovery	0.065	0.14	1.29.E−04	0.43 (0.28-0.67)	0.090	0.072
						Replication	0.071	0.15	1.39.E−03	0.43 (0.24–0.75)		
						Combined	0.067	0.14	6.64.E−07	0.43 (0.30–0.61)		
15	rs12594991	93516427	*CHD2*	intronic	A/G	Discovery	0.23	0.13	2.26.E−04	2.04 (1.42–2.94)	0.15	0.52
						Replication	0.24	0.11	1.40.E−04	2.56 (1.53–4.26)		
						Combined	0.24	0.12	2.03.E−07	2.21 (1.64–2.97)		

*AD* Alzheimer’s disease, *MAF* minor allele frequency, *OR* odds ratio, *CI* confidence interval, *SNP* single nucleotide polymorphism, *NA* not available.

^a^Physical position based on human reference genome build hg19 (GRCh37).

^b^The nearest gene to each SNP is underlined.

^c^*P* values were calculated using Cochran-Armitage trend test.

^d^Population frequency based on gnomAD database (http://gnomad.broadinstitute.org/).

### Novel variants associated with AD in the cAD chip application set

Candidate novel variants in the cAD chip application set were selected based on the following criteria: (1) only *P* values less than 0.005 were accepted; (2) MAF < 1% were excluded; and (3) common variants in non-Finnish European populations with a MAF value above 0.05 from the GnomAD database (http://gnomad.broadinstitute.org/) were removed; and (4) only the variants with the same direction of allelic effect, estimated using the odds ratio between the discovery set and validation set, were accepted. Subsequently, we identified nine AD-associated SNPs in eight genes (*CLIC4*, *PTPRN2*, *PSD3*, *SORCS1*, *LOC102724301*, *LINC01578*, *ABR*, and *USP32*) (Table 3). Six of these SNPs were not included in the IGAP consortium data because they were either not genotyped or were filtered out because of low MAF in European populations. Significant associations were not observed for the other three SNPs (rs12063304, rs967326, and rs79919241) in IGAP data. Notably, two intergenic SNPs found between *SORCS1* and *LINC01435* [rs144835823; *P* = 8.40 × 10^−^^4^, OR = 0.32 (95% CI: 0.15–0.65) and rs78442236; *P* = 9.57 × 10^−5^, OR = 0.17 (95% CI: 0.06–0.47)] and one intronic SNP in *USP32* [rs117665140; *P* = 8.17 × 10^−^^4^, OR = 1.62 (95% CI: 1.23–2.13)] were not found in European populations but were identified in our study. Taken together, we identified ethnic differences in associated single nucleotide polymorphisms (SNPs) at loci related to AD among different populations. We also identified 15 rare LOF variants such as frameshift, stop-gain, and stop-loss from the case-only or control-only categories of WGS analysis. However, none of these variants was significant in the cAD chip analysis.

**Table 3:** List of novel candidate variants significantly associated with customized genotyping AD chip application set (*P* value < 0.005).

Chromosome	SNP	Position^b^	Assigned genes^c^	Functional refGene	Minor/major allele	Our results					Known results^a^				
						Groups	MAF in cases	MAF in controls	*P* value^d^	OR (CI, 95%)	Non-effect/effect alleles	Beta (SE)^e^	*P* value	East Asian frequency^f^	European frequency (non-Finnish)^f^
1	rs12063304	25071668	*CLIC4*	Upstream	G/A	WGS	0.041	0.086	3.07.E−03	0.45 (0.26–0.78)	A/G	−0.023 (0.049)	0.64	0.063	0.029
						cAD chip	0.032	0.055	4.98.E−03	0.57 (0.38–0.84)					
						Combined	0.036	0.060	4.31.E−04	0.57 (0.42–0.78)					
7	rs80020083	158283748	*PTPRN2*	Intronic	A/G	WGS	0.038	0.077	8.67.E−03	0.47 (0.27–0.83)	NA	NA	NA	0.039	<0.001
						cAD chip	0.035	0.060	2.60.E−03	0.57 (0.39–0.83)					
						Combined	0.036	0.063	1.49.E−04	0.56 (0.41–0.76)					
8	rs967326	18547261	*PSD3*	Intronic	A/G	WGS	0.334	0.219	1.08.E−04	1.79 (1.32–2.42)	G/A	0 (0.034)	1.00	0.37	0.049
						cAD chip	0.338	0.287	4.35.E−03	1.27 (1.08–1.49)					
						Combined	0.336	0.276	4.90.E−05	1.33 (1.16–1.53)					
10	rs144835823	109002092	*SORCS1,LINC01435*	Intergenic	A/T	WGS	0.011	0.038	2.61.E−03	0.27 (0.11–0.68)	NA	NA	NA	0.022	0
						cAD chip	0.008	0.026	8.40.E−04	0.32 (0.15–0.65)					
						Combined	0.009	0.028	2.42.E−05	0.32 (0.19–0.56)					
10	rs78442236	109033178	*SORCS1,LINC01435*	Intergenic	A/G	WGS	0.008	0.036	1.10.E−03	0.21 (0.07–0.59)	NA	NA	NA	0.020	0
						cAD chip	0.004	0.022	9.57.E−05	0.17 (0.06–0.47)					
						Combined	0.005	0.024	1.99.E−06	0.21 (0.10–0.43)					
11	rs74352072	119792009	*LOC102724301,TRIM29*	Intergenic	G/C	WGS	0.245	0.166	4.95.E−03	1.63 (1.17–2.29)	NA	NA	NA	0.25	<0.001
						cAD chip	0.251	0.198	1.02.E−03	1.36 (1.13–1.62)					
						Combined	0.248	0.193	3.74.E−05	1.39 (1.19–1.61)					
15	rs79919241	93434633	*LINC01578*	ncRNA_intronic	T/C	WGS	0.172	0.104	6.17.E−03	1.80 (1.20–2.70)	C/T	0.049 (0.039)	0.21	0.066	0.043
						cAD chip	0.176	0.132	1.57.E−03	1.40 (1.13–1.73)					
						Combined	0.174	0.127	6.11.E−05	1.45 (1.21–1.73)					
17	rs201351606	1082940	*ABR*	Intronic	A/G	WGS	0.005	0.030	1.97.E−03	0.15 (0.04–0.55)	NA	NA	NA	0.026	<0.001
						cAD chip	0.005	0.018	1.80.E−03	0.25 (0.10–0.64)					
						Combined	0.005	0.020	3.37.E−05	0.23 (0.11–0.48)					
17	rs117665140	58280925	*USP32*	Intronic	T/C	WGS	0.100	0.038	9.28.E−04	2.77 (1.50–5.09)	NA	NA	NA	0.049	0
						cAD chip	0.099	0.063	8.17.E−04	1.62 (1.23–2.13)					
						Combined	0.099	0.059	7.73.E−06	1.74 (1.37–2.21)					

In the WGS, *n* = 500; cAD chip *n* = 1437; combined *n* = 1937.

*AD* Alzheimer’s disease, *MAF* minor allele frequency, *OR* odds ratio, *CI* confidence interval, *SNP* single nucleotide polymorphism, *NA* not available.

^a^Non-effect/effect alleles, Beta and *P* values in the International Genomics of Alzheimer’s Project (IGAP).

^b^Physical position based on human reference genome build hg19 (GRCh37).

^c^The nearest gene to each SNP is underlined.

^d^*P*-values were calculated using Cochran-Armitage trend test.

^e^Overall estimated effect size and standard error for the effect allele.

^f^Population frequency based on gnomAD database (http://gnomad.broadinstitute.org/).

rs1890078, previously observed in *APOE* ε4 carriers, was a common variant in *SORCS1*. However, rs144835823 and rs78442236 were very rare variants located approximately 24 kb and 55 kb away from the common variant, respectively. In the cAD chip application set, rs1890078 showed significant association in the *APOE* ε4 carriers but not in the *APOE* ε4- samples, whereas association of rs144835823 and rs78442236 with AD pathogenesis was observed, regardless of the *APOE* ε4 status. rs79919241, located in an intron of *LINC01578*, was approximately 82 kb away from rs12594991 in *CHD2*. The two variants were in low LD (*r*^2^ = 0.39) with each other, and were identified in *APOE* ε4 carriers (Table 2). rs12594991 (*P* = 2.03 × 10^−7^) exhibited a significantly higher association with *APOE* ε4 carriers than rs79919241 (*P* = 1.08 × 10^−^^4^). These results show the importance of performing *APOE* ε4 stratification analysis in case-control studies to enable the detection of significant variants related to the *APOE* ε4 allele in universal GWAS.

## Discussion

Large-scale GWAS such as the IGAP have reported various variants associated with AD; however, these studies have mainly focused on non-Asian populations, and the genetic architecture of AD is less clear in Asian populations. However, small-scale studies on the genetic diversity in AD across various ethnic groups have suggested the importance of understanding the genetic background of AD in diverse populations^29^. Since no large-scale genetic study of AD has been conducted in Koreans to date, this study sought to identify genetic variants associated with AD in a Korean population by using a large sample size. We discovered and validated genetic candidates associated with AD by using WGS data and a cAD chip dataset. Our findings provide an important perspective on AD pathogenesis related to the *APOE* ε4+ allele. The *APOE* ε4 allele, a well-known stratifying risk factor for AD, has been considered to primarily elucidate the genetic effects associated with AD pathogenesis. We suggest that a WGS approach is imperative to maximize the detection probability of the existence of population-specific or rare variants.

We detected two novel variants associated with AD in the *APOE* ε4 stratified analysis. Chromodomain helicase DNA binding protein 2 (*CHD2)* is characterized by the presence of a chromodomain that is responsible for chromatin remodeling. *CHD2* is a risk factor for photosensitivity in epilepsy^30^ and is related to neurodevelopmental disorders^31–33^. *CHD2* has previously been reported to interact with repressor element 1-silencing transcription factor (*REST*), which plays an important role in cognitive decline associated with AD^34^. The minor allele (A) confers risk for AD, affecting the gene expression of *CHD2* as cis-eQTL. According to the eQTL database^35^, one novel SNP (rs12594991) with minor allele (A) showing an association signal in the *APOE* ε4 carriers was significantly associated with high expression of the assigned gene (*CHD2*; FDR *P* < 0.001). The minor allele A on rs12594991 might be associated with high expression levels and increased susceptibility to AD. *CHD2* protein expression is high in the cerebral cortex and cerebellum, that is, in brain regions rather than other organs^36^ (The Human Protein Atlas; www.proteinatlas.org). Since sortilin-related VPS10 domain-containing receptor 1 (*SORCS1*) is associated with sortilin, *SORCS1* may be involved in amyloid precursor protein (*APP*) processing and trafficking across membranes, as is the sortilin-related receptor (*SORL1*) gene, which is associated with AD susceptibility^37^. Intronic genetic variations rs10884402 and rs950809 of *SORCS1* associated with late-onset AD have been reported in the Chinese Han population^38^. Interestingly, the intronic variants rs12571141, rs17277986, and rs6584777 of *SORCS1* only exhibited significant association in the *APOE* ε4 carriers^39^. However, the rs17277986 variants included in our cAD chip did not show significant associations in our dataset. Our novel variants in *SORCS1* are located in intergenic regions and show lower allelic frequencies than those of previously reported intronic variants. These results indicate that functional variants with biological implications are not always consistent with one another, despite being localized in the same gene, *SORCS1*.

We also identified putative novel variants that were not detected in European populations due to low allele frequency. Although our putative novel variants were selected by WGS analysis in the discovery stage with the *APOE* ε4 carriers, they were replicated in our cAD chip application set which was not stratified based on the *APOE* ε4 genotype. When we considered the *APOE* ε4 carriers in our cAD chip application set, four of these novel variants also showed significant association (*P* < 0.05) (Table 3). It is not known whether the four genes (*PTPRN2*, *SORCS1*, *LINC01578*, and *ABR*) containing the putative novel variants in the cAD chip dataset are directly involved in the pathogenesis of AD.

PH and SEC7 domain-containing protein 3 (*PSD3*) includes the putative Pleckstrin domain, indicating that *PSD3* is involved in intracellular signaling. *PSD3* was included in 30 top-scoring SNPs identified by a Bayesian combinatorial method in an AD GWAS dataset that was shown to be differentially overexpressed in AD^40^. Receptor-type tyrosine-protein phosphatase N2 (*PTPRN2*) could be involved in amyloid processing, based on the fact that *PTPRN2* is one of the substrates involved in beta-site APP-cleaving enzyme 1^41^. The expression of *PTPRN2* was significantly altered in the hippocampus of AD sufferers^42^. The active BCR-related gene (*ABR*), localized at the synapses of neurons, may be involved in synaptic signaling; the *ABR* gene is abundantly expressed in the brain^43^.

A number of limitations should be considered when interpreting our results. First, although we have conducted our association study with AD data from the largest Korean population size to date, it may still be inadequate. Therefore, novel variants identified in this study should be analyzed in a greater number of samples to achieve genome-wide significance. Second, the rare putative novel variants with low allele frequencies might not be replicated in European populations. We must validate these variants in other independent Asian populations.

In summary, our results highlight that novel germline variants associated with AD in *APOE* ε4 carriers sampled from a Korean population were identified using whole-genome sequencing and cAD chip genotyping. Our results suggest that genetic association studies must be performed in diverse ethnic populations.

## Supplementary information

Supplemental material

## Data Availability

The complete dataset will not be made publicly available because of restrictions imposed by the ethics committees due to the sensitive nature of the personal data collected. Requests for data can be made to the corresponding author.
