# Supplementary material for: Novel Alzheimer’s disease risk variants identified based on whole-genome sequencing of APOE ε4 carriers
Source: Transl Psychiatry. 2021 May 19;11:296. doi: 10.1038/s41398-021-01412-9 (PMC8134477; doi:10.1038/s41398-021-01412-9)
Supplement: Supplementary file 1 — Supplemental material [file 41398_2021_1412_MOESM1_ESM.docx]

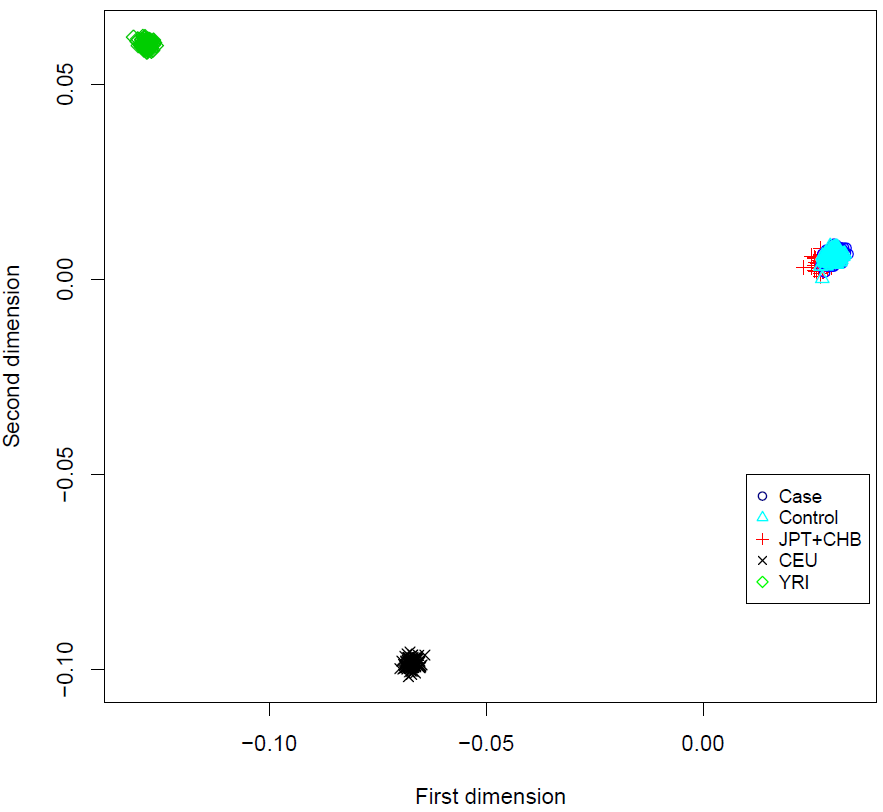


**Supplementary Fig. 1: Multi-dimensional scaling plot in the discovery set.** Multi-dimensional scaling analysis reveals that there is no population stratification in the discovery set. Case and control represent the 331 AD patients and 169 elderly controls used in the discovery set, respectively. Multi-dimensional scaling analysis was estimated for 270 individuals from the HapMap Project data. We used 90 individuals from the JPT + CHB population (Japanese in Tokyo and Han Chinese in Beijing), 90 from the CEU population (Utah Residents (CEPH) with Northern and Western European Ancestry), and 90 from the YRI population (Yoruba in Ibadan, Nigeria).


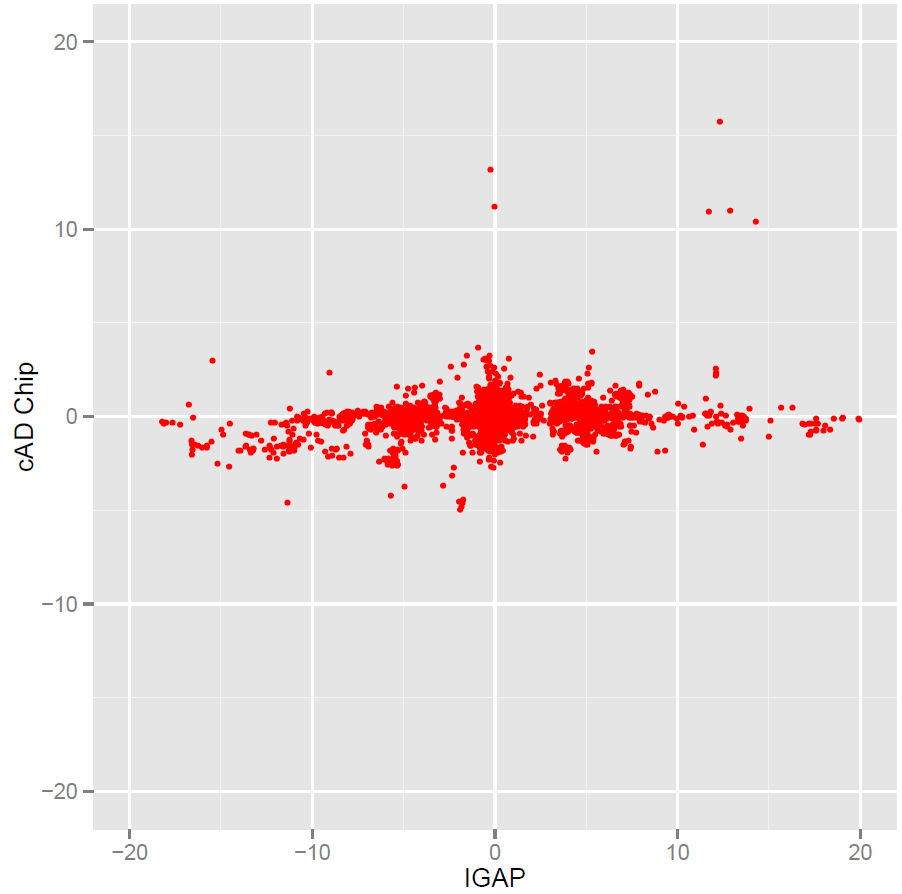


**Supplementary Fig. 2: Comparison of known variants reported from the International Genomics of Alzheimer's Project (IGAP) between Korean and European populations.** The scatter plot shows the distribution of *P* values adjusted in the direction of allelic effect in our cAD chip application set (n = 1437) and IGAP data. Y axis indicates –log_10_(P value) as association results in cAD application set encoding the direction of allelic effect as positive value for odds ratios greater than 1 and negative value for odds ratios less than 1. X axis indicates –log_10_(P value) from IGAP (stage 1 results) encoding the direction of allelic effect as positive value with more than beta 0 and negative value with less than beta 0. We only presented markers with less than –log_10_(P value) 20 from both results, respectively. Correlation was performed using Pearson’s correlation.

**Supplementary Table 1. Contents of customized genotyping AD chip.**

| **Source** | **Group** | **Criteria** | **Counts** |
| --- | --- | --- | --- |
| Database & Paper | AlzGene Database | Positive results | 1,448 |
|  | NHGRI-EBI GWAS catalog | Related with Alzheimer’s disease | 522 |
|  | IGAP Data | Stage 1 *P* < 0.00001 or Meta-analysis *P* < 0.00001 | 4,131 |
|  | *APOE* subgroup analysis result | Provided paper supplementary table | 1,127 |
|  | APP, PSEN1, and PSEN2 | dbSNP 147 and HGMD | 1,307 |
|  | In house Candidates | Additional candidates | 740 |
| WGS candidates | Case only group | Only discovered in Alzheimer’s disease samples | 2,357 |
|  | Control only group | Only discovered in control samples | 327 |
|  | Coding variant group | Variants from exonic regions (*P* < 0.05) | 1,396 |
|  | Non-coding variant group | Variants from noncoding regions (*P* < 0.001) | 29,606 |
|  | eQTL group | Variants related with eQTL | 570 |
| Total variants from database, paper, and WGS candidate | | | 42,480 |

**Supplementary Table 2. Clinical and demographic characteristics of study participants.**

| **Characteristics** | | **Discovery set (n = 500)** | | | **Validation set (n = 1,437)** | | |
| --- | --- | --- | --- | --- | --- | --- | --- |
|  |  | **Total** | **AD** | **Control** | **Total** | **AD** | **Control** |
| Number of samples | | 500 | 331 | 169 | 1437 | 543 | 894 |
| Gender, Female (%) | | 295 (59.0%) | 232 (70.1%) | 63 (37.3%) | 837 (58.2%) | 376 (69.2%) | 461 (51.6%) |
| Age^†^ | | 77 (73–82) | 78 (73–84) | 75 (73–79) | 75 (68–80) | 77 (70–82) | 73 (67–78) |
| Number of sample with *APOE* ε4 (%) | ε2/ε4 | 14 (2.8%) | 2 (0.6%) | 12 (9.8%) | 20 (7.0%) | 8 (4.2%) | 12 (12.4%) |
|  | ε3/ε4 | 440 (88%) | 286 (86.4%) | 107 (87.7%) | 210 (73.2%) | 129 (67.9%) | 81 (83.5%) |
|  | ε4/ε4 | 46 (9.2%) | 43 (13.0%) | 3 (2.5%) | 57 (19.9%) | 53 (27.9%) | 4 (4.1%) |

Abbreviations: AD, Alzheimer’s disease

^†^Age represents the median value. Ranges shown are inter-quartile ranges.

**Supplementary Table 3. Characterization of customized genotyping AD chip contents.**

| **Variant description** | **Number of contents in AD Chip** |
| --- | --- |
| Total variants | 42,480 |
| Autosome | 41,735 |
| X chromosome | 745 |
| Known variants (included in dbSNP 147) ^*^ | 40,395 |
| Novel variants (Not included in dbSNP 147) ^*^ | 2,085 |
| SNV | 40,367 |
| Small indel | 2,113 |

Abbreviations: AD, Alzheimer’s disease; SNV, single nucleotide variants

^*^Comparison based on dbSNP build 147

**Supplementary Table 4. Gene annotation of customized genotyping AD chip contents by using ANNOVAR.**

| **Precedence** | **Value** | **# SNV** | **# INDEL** | **# Total** |
| --- | --- | --- | --- | --- |
| 1 | exonic | 4,734 | 121 | 4,855 |
|  | splicing | 28 | 3 | 31 |
|  | exonic; splicing | 1 | 0 | 1 |
| 2 | ncRNA_exonic | 136 | 6 | 142 |
|  | ncRNA_intronic | 1,654 | 86 | 1,740 |
|  | ncRNA_splicing | 11 | 1 | 12 |
|  | ncRNA_exonic; splicing | 2 | 0 | 2 |
| 3 | UTR5 | 93 | 10 | 103 |
|  | UTR3 | 376 | 24 | 400 |
|  | UTR5;UTR3 | 1 | 0 | 1 |
| 4 | intronic | 13,408 | 747 | 14,155 |
| 5 | upstream | 284 | 17 | 301 |
|  | downstream | 280 | 22 | 302 |
|  | upstream; downstream | 18 | 1 | 19 |
| 6 | intergenic | 19,341 | 1,075 | 20,416 |
|  | Total | 40,367 | 2,113 | 42,480 |

**Supplementary Table 5. Two novel SNPs associated with APOE ε4 carriers did not show association with AD in *APOE* ε4 non-carriers (n = 1,150).**

| **Chromosome** | **SNP** | **Position^†^** | **Assigned Genes^¶^** | **Functional refGene** | **Minor/Major allele** | **MAF in Cases** | **MAF in Controls** | ***P* value^‡^** | **OR (CI, 95%)** |
| --- | --- | --- | --- | --- | --- | --- | --- | --- | --- |
| 10 | rs1890078 | 108978236 | SORCS1,LINC01435 | intergenic | C/T | 0.099 | 0.10 | 8.93.E-01 | 0.98 (0.73-1.33) |
|  |  |  |  |  |  |  |  |  |  |
|  |  |  |  |  |  |  |  |  |  |
| 15 | rs12594991 | 93516427 | CHD2 | intronic | A/G | 0.19 | 0.18 | 6.53.E-01 | 1.05 (0.84-1.32) |
|  |  |  |  |  |  |  |  |  |  |
|  |  |  |  |  |  |  |  |  |  |

Abbreviations: AD, Alzheimer’s disease; MAF, minor allele frequency; OR, odds ratio; CI, confidence interval; SNP, single nucleotide polymorphism; NA, not available

†Physical position based on human reference genome build hg 19 (GRCh37).

‡ *P*-values were calculated using Cochran-Armitage trend test

¶The nearest gene to each SNP is underlined.
